# Beyond Preconditioning: Postconditioning as an Alternative Technique in the Prevention of Liver Ischemia-Reperfusion Injury

**DOI:** 10.1155/2016/8235921

**Published:** 2016-06-02

**Authors:** Kassiani Theodoraki, Iosifina Karmaniolou, Aliki Tympa, Marios-Konstantinos Tasoulis, Constantinos Nastos, Ioannis Vassiliou, Nikolaos Arkadopoulos, Vassilios Smyrniotis

**Affiliations:** ^1^Department of Anesthesiology, Aretaieion University Hospital, University of Athens Medical School, 11528 Athens, Greece; ^2^Department of Anaesthetics, St George's Hospital, Blackshaw Road, London SW17 0QT, UK; ^3^Second Department of Surgery, Aretaieion University Hospital, University of Athens Medical School, 11528 Athens, Greece; ^4^Fourth Department of Surgery, Attikon University Hospital, University of Athens Medical School, 12462 Athens, Greece

## Abstract

Liver ischemia/reperfusion injury may significantly compromise hepatic postoperative function. Various hepatoprotective methods have been improvised, aiming at attenuating IR injury. With ischemic preconditioning (IPC), the liver is conditioned with a brief ischemic period followed by reperfusion, prior to sustained ischemia. Ischemic postconditioning (IPostC), consisting of intermittent sequential interruptions of blood flow in the early phase of reperfusion, seems to be a more feasible alternative than IPC, since the onset of reperfusion is more predictable. Regarding the potential mechanisms involved, it has been postulated that the slow intermittent oxygenation through controlled reperfusion decreases the burst production of oxygen free radicals, increases antioxidant activity, suppresses neutrophil accumulation, and modulates the apoptotic cascade. Additionally, favorable effects on mitochondrial ultrastructure and function, and upregulation of the cytoprotective properties of nitric oxide, leading to preservation of sinusoidal structure and maintenance of blood flow through the hepatic circulation could also underlie the protection afforded by postconditioning. Clinical studies are required to show whether biochemical and histological improvements afforded by the reperfusion/reocclusion cycles of postconditioning during early reperfusion can be translated to a substantial clinical benefit in liver resection and transplantation settings or to highlight more aspects of its molecular mechanisms.

## 1. Introduction

Prevention of major hemorrhage during hepatic resection is crucial because of the unfavorable short- and long-term outcomes associated with blood transfusion [1]. In this context, techniques involving some type of vascular control are favored by many surgeons since they can ensure a less hemorrhagic surgical field by taking advantage of liver tolerance to normothermic warm ischemia [2, 3]. These maneuvers, although valuable in preventing excessive blood loss, are invariably complicated by ischemia/reperfusion (IR) injury, which can reduce the capacity of the liver remnant to maintain adequate postoperative function [4, 5]. Hepatic IR injury can also occur in other clinical contexts, such as liver donor preservation and transplantation and hypovolemia [6, 7]. Specifically, both warm and cold ischemia, with the accompanying reduction of blood flow, cause depletion of hepatocyte energy reserves, accumulation of intracellular sodium, calcium, and reactive oxygen species (ROS), and the activation of multiple enzyme systems leading to cell damage [8]. With the restoration of blood flow through reperfusion, the liver is subjected to further injury secondary to an ensuing acute inflammatory response. Activated Kupffer cells, polymorphonucleocytes, and platelets infiltrate reperfused tissue, while further structural and functional disorders of hepatic tissue are mediated through abundant cytokine production, complement activation, accumulation of platelet activating factors and endothelial cell adhesion molecules, local imbalance in nitric oxide (NO) levels, and finally generation of free radicals and depletion of tissue antioxidant capacity [9–12]. The sequence of ischemia followed by reperfusion is manifested as vasoconstriction, neutrophil migration and adherence, and platelet aggregation [13–15]. The ensuing microcirculatory derangement can finally culminate in hepatocellular apoptosis and necrosis with untoward consequences not only for the liver but also for distant organs [16–18].

The extent of liver parenchymal damage depends on the duration of ischemia, the presence of preexisting liver disease, and the use of hepatoprotective methods. One of the methods used to modulate IR injury is ischemic preconditioning (IPC). IPC is the method by which the target organ is conditioned with a brief ischemic period followed by reperfusion prior to the subsequent prolonged ischemic insult in order to attenuate the extent of injury. Its beneficial effects were first reported by Murry et al. in a study of canine heart tissue [19]. It has since been adopted in liver surgery and tested in several experimental and clinical contexts, proving to be an effective intervention, since it seems to increase the ability of the liver to withstand the subsequent prolonged period of ischemia [20–23]. Adenosine and NO seem to play a significant role in the IPC effect and favorable responses such as decreased hepatocellular injury, inhibition of apoptosis, improved liver microcirculation, and enhanced energy metabolism have been documented through the application of IPC [24–27].

Regarding the clinical setting, in spite of favorable effects of enzyme markers of liver injury, recent meta-analyses failed to reveal a sustained clinical benefit of IPC, in terms of duration of hospital stay, perioperative morbidity, or mortality [28–30]. The main limitation of IPC techniques in the clinical context is that they must be initiated before the ischemic insult, which is not always predictable. In recent years, a novel approach to minimize IR injury was initiated. Ischemic postconditioning (IPostC), defined as several brief cycles of ischemia and reperfusion after the prolonged period of ischemia and before the sustained reperfusion was initially described by Zhao et al. in an in vivo canine heart model [31], was proved to provide effective cardioprotection with reduction of infarct size and reperfusion arrhythmias in subsequent studies [32–35] and its favorable effects were further confirmed in a meta-analysis including 123 patients [36]. The application of the conditioning stimulus (brief intermittent cycles of IR) after prolonged ischemia and prior to permanent restoration of blood flow, as opposed to IPC, where the conditioning stimulus is applied before the prolonged period of ischemia, makes the intervention clinically more relevant, since the onset of ischemia as already mentioned cannot always be predicted. Therefore, the technique was quickly adopted in experimental liver resection and transplantation settings and in recent years there have been several reports of its use in this context.

The aim of this review was to examine the current evidence for the use of IPostC in liver resection and transplantation settings, to establish whether IPostC protects against IR injury, and to provide some insight into the potential mechanisms involved.

## 2. Methods

We conducted a systematic PubMed® literature search for all types of published articles in the English language combining the free text and MeSH thesaurus terms: “liver”, “hepatectomy”, “liver transplantation”, “ischemia-reperfusion”, “ischemia-reperfusion injury”, “warm liver ischemia”, “cold liver ischemia”, “cold storage”, and “ischemic postconditioning”, “mechanical postconditioning”, and “pharmacological postconditioning”, in all possible combinations. Additional articles were found by manually cross-checking the references of the identified articles. In total, 25 articles pertinent to postconditioning were retrieved. Specifically, 19 studies on ischemic (mechanical) postconditioning and six studies on pharmacological postconditioning were found relevant and suitable for inclusion in the present review. Articles highlighting the specific mechanisms of postconditioning were also considered.

## 3. Mechanical (Ischemic) Postconditioning

In recent years, several experimental IR injury models have been described using various ischemic and reperfusion times in order to test the efficacy of postconditioning maneuvers (Table 1). The first study which demonstrated the protection afforded by IPostC on liver injury was reported by Sun et al., in a rat model of ischemia and reperfusion [37]. The authors established a 70% hepatic ischemia model by occluding blood flow to the left and caudate lobes of the liver. Ischemia was maintained for 60 min, followed by a 120-minute reperfusion period. In the conditioned group, IPostC consisted of several brief prereperfusions (2, 3, 5, and 7 min consecutively, each separated by occlusion time of 2 min), followed by the sustained reperfusion. Concentrations of malondialdehyde (MDA), as an index of tissue lipid peroxidation, and activities of several antioxidant enzymes (superoxide dismutase (SOD), catalase (CAT), and glutathione peroxidase (GSH-P_X_)) were measured in hepatic tissue. Additionally, apoptotic cells were measured by terminal deoxynucleotidyl transferase dUTP nick end labeling (TUNEL) staining, expression of Bcl-2 protein (which is a major representative of apoptotic restraining proteins) was measured by immunohistochemical techniques, and mitochondrial ultrastructure was evaluated by electronic microscope. In comparison to the IR group, the concentration of MDA in the IPostC group was markedly reduced while the activity of all antioxidant enzymes and the expression of Bcl-2 protein were significantly enhanced. Moreover, the apoptotic index in the IPostC group was significantly reduced and mitochondrial ultrastructure was maintained basically normal, with attenuation of mitochondrial swelling and maintenance of the integrity of outer mitochondrial membrane. In a similar model of controlled reperfusion, Zhang et al. additionally measured alanine aminotransferase (ALT) and aspartate aminotransferase (AST) as well as nuclear factor-kappa beta (NF-*κ*B) p65 (whose generation is activated by a large amount of oxygen free radicals) and also found lower levels of ALT and AST, increased SOD activity, and reduced NF-*κ*B p65 expression and apoptotic index in the IPostC group in comparison to the IR group [38]. They also observed differences in light and electron microscopy, with a lower degree of congestion of hepatic sinusoids, reduced neutrophilic infiltration, and fewer cells with disruption of nuclear and mitochondrial membranes or degranulation of endoplasmic reticulum in the IPostC group, as compared to the IR group. The authors attributed the protection afforded by postconditioning to the controlled slow intermittent oxygenation through the several cycles of on/off flow before permanent reperfusion. Finally Yoon et al., adopting the same sequence of on/off flow before permanent reperfusion, showed increased expression of the antiapoptotic protein survivin in the IPostC group in comparison to the IR group [39].

Different protocols of postconditioning have been used by other authors. Teixeira et al., using a rapid intermittent reperfusion consisting of five periods of 5 sec of opening followed by 5 sec of clamping each before the prolonged reperfusion, demonstrated decreased lipid peroxidation assessed by MDA levels and enhanced expression (although not significantly) of the glutathione-s-transferase-*α*-3 gene, assessed by real-time RNA analysis [40]. Also, by interposing three cycles of reperfusion followed by three cycles of ischemia (lasting 30 sec each) between ischemia and prolonged reperfusion, another group of researchers showed attenuated liver injury assessed by serum AST and ALT levels and attenuated histological scores of hepatic lesion in the postconditioned group [41]. Lin et al., after generating a 45-minute period of left lobe ischemia, performed three cycles of 1-minute reperfusion followed by 1 min of ischemia before sustained reperfusion and measured serum ALT levels and the degree of apoptosis by TUNEL staining and the formation of 4-hydroxy-2-nonenal (4-HNE) modified protein, which is generated during the lipid peroxidation cascade and is used as a biomarker of oxidative stress. They also evaluated cytochrome c release by mitochondria with Western blot analysis and change of mitochondrial membrane potential through flow cytometry analysis [42]. In comparison to the IR group, IPostC attenuated the elevation of ALT as well as the apoptotic count and decreased lipid peroxidation, as assessed by decreased expression of 4-HNE protein. IPostC also effectively reduced cytochrome c release from mitochondria and preserved mitochondrial membrane potential as compared to nonconditioned animals. Based on the notion that the opening of mitochondrial permeability transition pores (mPTP) with the resultant release of cytochrome c and collapse of transmembrane potential plays a crucial role in the pathogenesis of reperfusion injury, the authors concluded that the hepatoprotective effects of postconditioning are mediated through modulation of mitochondrial permeability transition.

Most of the studies mentioning histological results have based their conclusions on semiquantitative histopathologic evaluations. A recent study used stereological methods to obtain quantitative three-dimensional histological information in order to assess the effect of IPostC on the liver [43]. In this report, Knudsen et al. focused on necrosis and apoptosis which are irreversible signs of hepatocellular injury in contrast to vacuolization and sinusoidal congestion, which may be reversible, and additionally based their results on design-based stereology, which is an objective and highly reproducible method [44]. The authors established their ischemic model by interrupting portal trial flow to the median and left lobes for 60 min after which they evaluated a 4- or 24-hour reperfusion period. The IPostC protocol consisted of three cycles of 30 sec of reperfusion and 30 sec of ischemia performed immediately after the 60 min of prolonged ischemia. Although no significant differences were demonstrated after four hours of reperfusion, at 24 hours of reperfusion, the authors observed a significant decrease in necrotic volume ratio (NVR) and an insignificant decrease of apoptotic cell profiles in the IPostC group in comparison to the control group.

IPostC has also been evaluated in the experimental context of liver transplantation. In a rat model of orthotopic liver transplantation, Wang et al. applied postconditioning after cold ischemia of the donor liver using several intermittent interruptions of blood flow at the early phase of reperfusion and compared grafts treated with IPostC with controls, after six hours of sustained reperfusion of the liver graft [45]. Apart from standard histopathologic examination, they evaluated serum parameters of hepatocellular injury (AST, ALT, and lactate dehydrogenase (LDH)), lipid peroxidation (MDA), antioxidant enzyme activity in liver tissue (SOD, GSH-P_X_), and myeloperoxidase (MPO) as a marker of polymorphonuclear neutrophil infiltration. They also used reverse transcriptase-polymerase chain reaction (RT-PCR) RNA analysis to quantify the expression of tumor necrosis factor-alpha (TNF-*α*) and macrophage inflammatory protein-2 (MIP-2) in liver tissue. They additionally measured NO content in serum and the expression of inducible NO synthase (i-NOS) and endothelial NO synthase (e-NOS) in liver tissue. Markers of hepatocellular injury were markedly reduced when grafts were treated with postconditioning, while IPostC inhibited lipid peroxidation, enhanced antioxidant enzyme activity, suppressed polymorphonuclear accumulation, lowered the expression of TNF-*α* and MIP-2, and reduced the extent of necrosis in histopathologic examination. Furthermore, increases in serum NO and i-NOS and e-NOS expression were much more prominent when grafts were treated with postconditioning. The authors postulated that i-NOS- and e-NOS-mediated endogenous NO production might also underlie the protection afforded by IPostC. Zeng et al. also demonstrated that IPostC attenuated liver IR injury in a cold ischemia model, since it attenuated serum transaminase levels and histological damage in postconditioned grafts, while it reduced MDA production and increased SOD activity in comparison to the IR group [46]. They implicated induction of the cytoprotective enzyme heme oxygenase-1 (HO-1) in the enhancement of antioxidative activity associated with postconditioning. They reinforced their findings in a subsequent study, where, by pretreating donors with an inhibitor of HO-1 before liver harvest, they demonstrated negation of the protective effects of IPostC [47].

The only clinical study on IPostC has just recently been reported [48]. The authors attempted to test the role of IPostC in human liver transplantation and its effects on IR injury and liver graft function. IPostC consisted of three 1-minute cycles of reperfusion interspersed with three 1-minute cycles of arterial occlusion immediately after arterial reperfusion of the graft. Median postoperative peak AST values, indices of early graft dysfunction, apoptosis, morbidity (including graft rejection), early postoperative mortality, and one-year patient and graft survival were similar between patients subjected to IPostC and the control group. However, grafts subjected to postconditioning presented less severe histopathological lesions of IR injury and increased activation of autophagy in periportal areas. Autophagy is triggered by stress conditions to ensure cell survival by restoring adequate levels of intracellular ATP, a fact that has been correlated with better posttransplantation outcomes [49, 50]. Therefore, the study results showed that grafts subjected to IPostC had better tolerance of IR injury according to histological parameters, which, together with the induction of autophagy, is indicative of the restoration of sufficient energy reserves in injured hepatocytes.

## 4. Pharmacological Postconditioning

Apart from mechanical postconditioning, there have been studies showing that some agents can be used as pharmacological inducers of postconditioning. The administration of agents upon liver reperfusion aiming at mitigating IR injury is defined as pharmacological postconditioning (Table 2). Milrinone, a phosphodiesterase-3 inhibitor, is an inotropic agent, acting through elevation of intracellular cyclic adenosine monophosphate (cAMP) and protein kinase A (PKA) activation. It has been shown that it also has preconditioning properties against hepatic IR injury, exerted via the same pathway (cAMP/PKA activation) [51]. Toyoda et al., in a liver warm ischemia model of one-hour duration followed by five hours of reperfusion, showed that milrinone administered as an intravenous bolus immediately after reperfusion effectively attenuated liver injury, as demonstrated by reduced AST, ALT, and LDH serum levels and reduced histologic damage and apoptotic scores in milrinone-treated animals, as compared to controls [52]. The authors postulated that the protective effect of milrinone could be mediated through phosphatidylinositol 3-kinase (PI3K-Akt) and NOS activation, as the beneficial effects of milrinone were abrogated by inhibition of PI3K-Akt and NOS.

Tian et al. administered diazoxide (a selective ATP-dependent mitochondrial potassium (mito-K_ATP_) channel opener) before reperfusion, in a warm liver ischemia rat model [53]. Diazoxide treatment significantly attenuated hepatic injury, as demonstrated by serum levels of AST and ALT, while it upregulated levels of protein kinase c-epsilon (pkc-*ε*), which is a kinase necessary for the opening of mito-K_ATP_ channels that are involved in the protection from reperfusion injury [54, 55]. Additionally, diazoxide inhibited the activation of the apoptotic pathway by increasing the expression of apoptotic restraining protein Bcl-2 and by decreasing the release of cytochrome c and the expression of caspase-3.

Beck-Schimmer et al. designed the only known clinical study of pharmacological hepatic postconditioning, using the anesthetic agent sevoflurane [56]. They evaluated whether pharmacological postconditioning with sevoflurane confers protection during liver surgery under inflow occlusion compared with control and whether pharmacological postconditioning with sevoflurane confers equivalent protection to the technique of intermittent clamping during ischemia. All patients were anesthetized with propofol. In the postconditioning group, sevoflurane was administered for a 30-minute period upon reperfusion of the liver, replacing propofol infusion. The postconditioning group displayed lower peak AST values within the first seven postoperative days as well as shorter hospital stay and a reduced risk of complications in comparison to the control group. No significant differences were demonstrated between the postconditioning and intermittent clamping groups, indicating a similar degree of protection. The same group, in a previous study, had demonstrated that pharmacological preconditioning with sevoflurane provided hepatoprotection in patients undergoing major liver resection [57]. The importance of these studies lies not only in the fact that they were performed on human subjects but also in the fact that reduced laboratory indices of liver injury were accompanied by improvements in clinical outcome.

Dal Ponte et al. investigated whether an adenosine A_2_A receptor agonist could act as a pharmacological inducer of postconditioning [58]. They set up an in vitro experimental model of freshly isolated rat hepatocytes mimicking hepatocyte reoxygenation injury after the cold ischemia phase of liver graft preservation. The addition of the A_2_A receptor agonist significantly reduced hepatocyte death upon reoxygenation through a PI3-Akt-mediated response. They further confirmed their results in an in vivo model of warm IR injury, where rats exposed to intraperitoneal injection of the A_2_A receptor agonist immediately upon reperfusion presented with reduced ALT release and fewer necrotic areas on histological examination, as compared to controls. The authors concluded that adenosine A_2_A receptor stimulation effectively elicits postconditioning responses in liver cells through modulation of PI3-Akt-dependent signaling and is in agreement with studies that have shown that the stimulation of the same receptors triggers hepatic preconditioning and prevents cell death [59, 60].

Ginsenoside Rb1 (Rb1) is the effective ingredient of ginseng root, a root with known antioxidant properties. There have been reports of favorable effects of Rb1 on liver injury induced by intestinal IR or tert-butyl hydroperoxide [61, 62]. Guo et al. demonstrated its postconditioning effects in a mouse warm liver ischemia model [63]. They showed reduced serum ALT levels and lower scores of cytoplasmic vacuolization, sinusoidal congestion, and hepatocyte necrosis on histological examination as well as suppression of the overexpression of proinflammatory mediators and adhesion molecules, decreased concentration of MDA, and increased activity of SOD in hepatic tissues of Rb1-postconditioned animals. They suggested that the protection conferred by Rb1 appears to be NO-mediated, as serum levels of NO and NOS and expression of NOS in liver tissues were also increased compared to controls.

Finally, Shawky et al. investigated the effect of intraportal recombinant human erythropoietin (rhEPO) in a rat model of hepatic IR injury as well as its appropriate time and dose of administration [64]. Moreover, they compared preconditioning with rhEPO (24 h or 30 min before ischemia) with postconditioning with rhEPO (administering it at reperfusion). Both preconditioning and postconditioning with rhEPO were effective in attenuating hepatic injury, as assessed by decreased AST and ALT serum levels while preconditioning was more effective than postconditioning in attenuating IR-induced apoptosis, as assessed by the reduction of caspase-9 activity and the increase of antiapoptotic Bcl-xL/apoptotic Bax ratio.

## 5. Comparison between Postconditioning and Preconditioning

A few comparative studies between IPC and IPostC have also been undertaken. Zhang et al. demonstrated equal protection by IPC and IPostC in terms of AST, ALT, and SOD activity, apoptotic index, and light and electron microscopy findings [38]. Wu et al., using a rat model of segmental IR injury, showed that the level of protection afforded by postconditioning is comparable to that afforded by preconditioning regarding production of ROS, maintenance of the activity of the antioxidant systems, suppression of neutrophil recruitment, and animal survival [65]. A comparative study between IPC and IPostC has also been reported by Wang et al. [66]. The authors established a warm IR hepatic model, by clamping the hepatic pedicle for 30 min, and a cold IR model, by performing orthotopic liver transplantation within two hours of cold storage. IPC and IPostC provided an equal level of protection in terms of serum transaminase levels, biliary epithelial cell function, hepatic morphology, survival rate, and the expression of Fas apoptotic gene. Interestingly, in the cold ischemic model, IPostC was more effective than IPC in inhibiting apoptosis, as expressed by a lower apoptotic index. In a murine warm hepatic IR model, Song et al. demonstrated that pre- and postconditioning protocols were equally effective in reducing liver injury as assessed by reduction of AST and ALT levels, suppression of cytokine and MDA levels, and increase of the activity of antioxidant enzymes (SOD, CAT, GSH-P_X_, and NOS) [67]. Additionally, both IPC and IPostC upregulated the expression of hypoxia inducible factor 1-alpha (HIF-1*α*) and of vascular endothelial growth factor (VEGF). HIF-1*α* is a master transcriptional factor activated by low oxygen tension that facilitates cellular adaptation to hypoxia in oxygen-deficient environments [68] and it can activate other genes, such as VEGF. VEGF acts on endothelial cell proliferation, migration, and cell organization during recovery phases after hepatic microvascular dysfunction, promoting the secretion of growth and survival factors [69]. In the study by Knudsen et al., who used design-based stereology to obtain quantitative three-dimensional histological information, both IPC and IPostC were equally effective in preventing hepatocellular necrosis, as assessed by NVR, while a significantly lower number of apoptotic cell profiles were achieved only in the IPC group [43]. In contrast, the number of apoptotic cell profiles decreased insignificantly in the IPostC group. Finally, Shawky et al. compared preconditioning and postconditioning with rhEPO, administered before ischemia and immediately after reperfusion, respectively [64]. As already mentioned, they demonstrated that preconditioning was more effective than postconditioning in attenuating IR-induced apoptosis, as assessed by the reduction of caspase-9 activity and the increase of antiapoptotic Bcl-xL/apoptotic Bax ratio.

## 6. Combination of Preconditioning and Postconditioning

IPC and IPostC have been combined in a couple of studies. Song et al. tested the combination of IPC and IPostC in the murine warm hepatic IR model described previously [67]. The combination of IPC and IPostC offered synergistic protection in comparison to IPC or IPostC alone, decreasing AST and ALT levels and increasing the activity of antioxidants, of hypoxia tolerance response, and of the speed of cell proliferation at a greater extent as compared to the individual treatments. However, the combination had no additional favorable effect on cytokine release as compared to solo treatment, probably because, by individual application of IPC and IPostC, the limit of decreased cytokine release is reached, as suggested by the authors [67]. The second study in which the combination of IPC and IPostC was evaluated was the work by Wu et al. [65]. The authors demonstrated equal protection of the combined treatment in comparison to IPC or IPostC alone on the production of ROS, the maintenance of the activity of the antioxidant systems, and the suppression of neutrophil recruitment. However, in that study, no additive effect from the combined treatment on the reduction of liver IR injury was demonstrated.

## 7. Postconditioning and Liver Regeneration

In the study by Song et al. [67], both IPC and IPostC upregulated the expression of HIF-1*α* and of VEGF, which is a factor crucial for endothelial cell proliferation and organization during tissue recovery phases, as mentioned above. However, in another study, HIF-1*α* did not prove to be a mediator of the cytoprotective effects of either preconditioning or postconditioning, since HIF-1*α* mRNA expression was lower in all conditioned groups in comparison to the IR group [70]. Similarly, conditioning did not upregulate the expression of VEGF. In the same study, no significant differences in ALT serum levels were demonstrated between conditioned groups and the IR group. However, as the authors themselves acknowledge, these findings could be explained by the fact that they used a very short period of ischemia and reperfusion (30 min resp.), which might not be adequate to demonstrate the full extent of IR injuries. Therefore, they were unable to see any hepatoprotective effects of conditioning, as assessed by changes in liver parameters, as these have been demonstrated in other studies. The very short period of follow-up after reperfusion could also explain the low levels of HIF-1*α* and VEGF in preconditioned and postconditioned animals in their model, as the 30 minutes of reperfusion studied and the lack of following expression levels over time probably did not allow the full effect of changing HIF-1*α* and VEGF levels to develop, according to the authors [70]. Additionally, Knudsen et al. studied the effect of ischemic pre- and postconditioning on the expression of genes with angiogenic potential in rat liver [71]. In the conditioned groups, genes involved in angiogenesis were significantly upregulated. Therefore, both preconditioning and postconditioning seem to be potent activators of angiogenic genes. However, as the authors themselves state, this might prove a double-edged sword, since it may prove favorable for the regenerating liver but on the other hand it might stimulate the growth of micrometastases [72, 73]. The same group of authors, in a subsequent study aiming to investigate the genomic response induced by IPC or IPostC, used the same experimental protocol and validated their microarray analysis by performing quantitative real-time PCR [74]. They found that a substantial number of genes, especially those involved in DNA binding and transcription, cellular membrane function, apoptosis, and metabolic processes are affected, especially by postconditioning or by the combination of preconditioning and postconditioning. This indicates that conditioning techniques might mediate their protective effect during the early reperfusion phase by activating the expression of gene networks crucial to cellular growth, proliferation, repair, and homeostasis. Overall, upregulated pathways seem to increase the cellular resistance to stressful conditions and the defense of the rat liver against IR injuries.

Finally, the most recent study investigating the effect of postconditioning or preconditioning on liver regeneration is by Young et al., who subjected prepubertal rats to total hepatic ischemia for 30 min through hepatic pedicle clamping and then to 24-hour reperfusion [75]. IPC consisted of 10-minute ischemia followed by 10-minute reperfusion before the main ischemic event and IPostC consisted of two 30-second reperfusion/clamping cycles before sustained reperfusion. The authors measured AST and ALT serum levels, proliferating cell nuclear antigen (PCNA) with immunohistochemistry, and evaluated liver regeneration with the calculation of regenerated liver mass according to a special formula. They found that both IPC and IPostC attenuated AST and ALT levels and thus protected the liver of growing rats against IR injury and that, interestingly, IPostC was more effective than IPC in terms of liver regeneration. This is in contrast with a couple of studies which demonstrated increased cell proliferation in models of total ischemia and partial hepatectomy with IPC; however both these studies used extracorporeal shunts and probably this should be taken into account [76, 77]. As the authors state, their findings could prove of great interest in the context of split-liver transplantation or living donor liver transplantation, where there is subsequent need for regeneration of both donor remnant and recipient graft liver and therefore the protection afforded by IPostC could offer an additional advantage.

## 8. Mechanisms of Hepatic Postconditioning

The exact mechanism through which postconditioning exerts its protective action on liver tissue has not been elucidated yet. It has been postulated that the slow intermittent oxygenation, when controlled reperfusion is applied through postconditioning, decreases the burst production of oxygen free radicals that accumulate with abrupt oxygenation of the liver cell and thus stimulates the release of intracellular antioxidant enzymes and free radical scavengers that convey hepatoprotection. Lower levels of lipid peroxidation substances, as expressed by MDA, and higher levels of antioxidant enzymes induced by IPostC in the aforementioned studies show its ability to act as a line of defense against oxidative injury in tissues [37, 38, 45–47, 65, 67]. MDA is a cytotoxic reactive aldehyde, which is formed when cell membranes are degraded by ROS; therefore it is considered as a biomarker of oxidative stress [78]. Pharmacological postconditioning also may reduce oxidative stress by attenuating the increase of MDA and increasing antioxidant activity [63].

Although the enormous generation of ROS during reperfusion plays an important role in reperfusion injury, low levels of ROS have been paradoxically implicated as an essential transduction component in protective pathways, upregulating the endogenous antioxidant enzyme activities, a fact referred to as “redox signaling.” Therefore, moderate concentrations of ROS during reperfusion might also confer a beneficial effect and ROS signaling at early reperfusion has been shown to mediate protective effects of both IPC and IPostC in heart tissue [79, 80]. It could thus be possible that one of the triggers of hepatic postconditioning protection could be the ROS availability during early reperfusion, which might contribute to the activation of protective intrinsic mechanisms against the deleterious effects of the subsequent reperfusion.

Additionally, it has been postulated that cell apoptosis could also be a primary mechanism of the damage evoked by liver IR injury [17, 18]. Modulation of the apoptotic cascade has been demonstrated in a variety of postconditioning studies and together with downregulation of the release of proapoptotic solutes could be one aspect of the protection afforded by IPostC [37, 38, 42, 66]. Pharmacological inducers of postconditioning were also found to inhibit the activation of the apoptotic pathway [52, 53, 64]. In relation to oxidant-induced apoptosis, the role of NF-*κ*Β has also been investigated. NF-*κ*Β is a transcriptional factor whose generation is activated by a large amount of oxygen free radicals [81]. It has been shown that oxidant-induced apoptosis after myocardial ischemia and reperfusion can be activated through translocation of NF-*κ*Β and stimulation of the release of ΤNF-*α* while application of postconditioning at the onset of reperfusion attenuated myocardial apoptosis through inhibition of NF-*κ*Β translocation [82]. Reduced NF-*κ*B p65 expression along with apoptotic index in the IPostC group in comparison to the IR group was demonstrated in the Zhang study [38]. Therefore, hepatic postconditioning could also be associated with the inhibition of oxidant-mediated activation of nuclear factor *κ*Β-TNF-*α* signaling pathway.

Apart from improving activity of endogenous antioxidant enzymes, postconditioning could exert its protective effect by inhibiting neutrophil accumulation, as IPostC has been shown to suppress neutrophil infiltration in hepatic tissues as well as chemokines that play a crucial role in neutrophil recruitment and activation, like MIP-2 [45]. IPostC has also been demonstrated to suppress the expression of intercellular adhesion molecule-1 (ICAM-1) in liver tissue [83], while suppression of ICAM-1 has also been demonstrated by Rb1 postconditioning [63]. ICAM-1 is one of cell-surface adhesion molecules that are known to mediate leukocyte-endothelial cell interaction. This molecule is present at low levels on most endothelial cells and is upregulated in case of inflammation and IR injury [84]. In fact, blocking its activity with monoclonal antibodies has been found to protect against IR injury [85, 86]. Accumulated polymorphonucleocytes act as important effector cells in the pathogenesis of IR liver injury. During ischemia, neutrophils accumulate in the endothelium and such accumulation may be markedly accelerated following reperfusion. Activated neutrophils release a variety of cytotoxic substances interacting with the endothelium and thereby causing tissue damage and releasing large amounts of ROS, which contribute to the oxidative injury associated with hepatic IR injury [87, 88]. Furthermore, it has been shown that Kupffer cell stimulation might lead to excessive production of TNF-*α* at the initial phase of reperfusion [89]. TNF-*α* could stimulate chemokine production like MIP-2 in hepatocytes of prolonged IR injury, which in turn facilitates polymorphonuclear activation [90]. It could thus be possible that postconditioning might protect from reperfusion injury through downregulation of substances that mediate neutrophil adhesion and activation like TNF-*α*, MIP-2, and ICAM-1, as it has been shown in some of the aforementioned studies [45, 63, 83].

It has also been suggested that the hepatoprotective effects of postconditioning are mediated through favorable effects on mitochondrial ultrastructure and function [37, 38, 42]. It has been postulated that the burst of oxygen free radicals generated by reperfusion reacts with unsaturated fatty acids on the surface of mitochondrial membrane, leading to opening of mitochondrial permeability transition pores (mPTP) [91]. When these pores open, the electrochemical gradient across the inner mitochondrial membrane is disrupted, which results in swelling and rupture of the outer mitochondrial membrane [92]. Release of apoptosis-related substances originally located in the mitochondria including cytochrome c ensues, which then transfer into the cytoplasm and subsequently activate the downstream cascade apoptosis reaction, while at the same time playing a key element in cell death [93–96]. Administration of mitochondrial permeability transition inhibitors has been shown to mitigate reperfusion injury after experimental liver transplantation [97]. Targeting mitochondrial dysfunction and inhibition of mPTP could also underlie the hepatoprotective mechanism of postconditioning. In fact, in IPostC studies, electron microscopic imaging has revealed attenuation of mitochondrial damage and basically intact mitochondrial membranes in animals subjected to the slow controlled reperfusion through postconditioning, while mitochondrial swelling and disrupted mitochondrial membranes were evident in livers not subjected to postconditioning [37, 38, 47]. In these studies, manifestations of apoptosis like condensation of chromatin into clumps at the edge of the nucleus and swelling and rounding of cells were also evident by electron microscopic observation. Additionally, Lin et al. demonstrated effective reduction of cytochrome c release from mitochondria by IPostC as well as preservation of mitochondrial membrane potential [42]. Decreased expression levels of cytochrome c in hepatic tissue were also demonstrated when diazoxide was used as a pharmacological inducer of postconditioning [53]. Therefore, inhibition of mPTP opening could be associated with the cytoprotective effects of postconditioning [98–100]. However, the real trigger that inhibits mPTP opening by postconditioning is not known. It has been postulated by Cohen et al. that IPostC maintains intracellular acidosis at the initial stage of reperfusion and that perpetuation of acidosis inhibits mPTP opening in cardiac IR models [101]. The pH hypothesis though and its applicability in liver tissue need to be validated by further studies. With relevance to the mitochondria, the opening of mito-K_ATP_ channels could also underlie the protection of hepatic postconditioning. Activation and translocation of pkc-*ε*, which consequently facilitates the opening of mito-K_ATP_ channels, have been shown to be crucial in triggering the cardioprotective effects of IPC and IPostC [54, 55, 102, 103] and were shown to be the mechanism by which diazoxide exerts its hepatic postconditioning effect [53].

NO could also underlie the protection afforded by postconditioning. In fact, it has been shown that increases in NO serum content and upregulation of NOS expression are much more prominent in IPostC treated animals; therefore, it has been postulated that e-NOS- and i-NOS-mediated NO production may be an important mechanism of this protection [45, 83]. NO is considered a controversial mediator of physiological and pathological processes inherent in IR injury since it has been shown to have both protective and deleterious effects on cellular function [104]. In fact, several studies that have investigated the role of NO in partial liver ischemia-reperfusion models have provided controversial results [11, 104, 105]. NO is synthesized from L-arginine by three isoforms of the NOS, the e-NOS, the i-NOS, and the neuronal synthase [105]. Whether NO has a protective or deleterious effect probably depends on the source and quantity of NO produced and the cellular redox status of the liver [105–107]. On the one hand, NO stimulation under oxidative stress conditions can induce reperfusion-mediated liver injury through lipid peroxidation, DNA damage, and proapoptotic effects [105]. In the presence of superoxide, NO forms peroxynitrite, a potent oxidant agent which can decompose to generate an extremely hepatotoxic substance [108]. Therefore, apart from favorable actions, excessive production of NO may also prove deleterious and have cytotoxic potential through its interaction with superoxide anion and contribute to the hepatic injury evident in the late phases of reperfusion.

On the other hand, e-NOS-derived NO is considered to have a cytoprotective effect in IR injury, playing an important role in regulation of intracellular calcium levels and inhibition of platelet aggregation and counteracting the vasoconstriction caused by endothelin, particularly during the early stages of liver IR [9, 105, 109]. e-NOS expression is downregulated during liver reperfusion as a result of inhibition of e-NOS activity by oxidative stress and absence of flow within the sinusoids during ischemia [104]. The decreased production of NO from e-NOS increases the vascular resistance of the intrahepatic circulation and contributes to the microcirculatory dysfunction following reperfusion [14, 110]. It has been shown that IR injury is exacerbated in e-NOS- and i-NOS-deficient animal models [111, 112], whereas genetic overexpression of e-NOS has been shown to attenuate hepatic IR injury in a rat model [113]. NO production by e-NOS has also been shown to act favorably in renal and myocardial IR injury [114, 115]. Therefore, enhanced e-NOS expression has a cytoprotective effect and acts protectively by preservation of the sinusoidal structure and maintenance of blood flow through the hepatic microcirculation [104]. Favorable effects of inhaled NO in the clinical context of liver injury have also been reported, since it was shown to accelerate restoration of liver function in adults after liver transplantation [116].

Additionally, it has already been suggested that the protective role of IPC in the liver is mediated through NO pathways [24, 117, 118] while there is evidence implicating NO in the protection of IPostC in tissues other than the liver [110, 119]. Therefore, the protective effect of IPostC in liver IR injury could similarly be related to the enhanced level of expression of e-NOS and i-NOS, which in turn increase endogenous NO production [45, 83]. Additionally, milrinone-induced postconditioning could be mediated through NOS activation, as a NOS inhibitor, injected before milrinone administration, completely abrogated the protective effects of milrinone, in the study by Toyoda et al. [52]. The postconditioning effect of Rb1 was also found to be mediated through NOS activation and increased NO production in serum and liver tissues, in the study by Guo et al. [63]. Upregulated NO by IPostC or pharmacological postconditioning might also have a role in modulating the inflammatory process by downregulating the expression of TNF-*α* and ICAM-1 [63, 83]. In fact, NO has been reported to decrease ICAM-1 expression, which results in reduction of polymorphonucleocyte adhesion to the endothelium stimulated by TNF-*α* [120]. Additionally, NO donors have been found to attenuate leukocyte-endothelial cell reactions and adhesive interactions, thus maintaining vascular patency [121].

The exact mechanisms through which enhanced levels of NO when postconditioning is applied convey hepatoprotection are yet to be defined. Guo et al. demonstrated increased expression of HIF-1*α* and PI3K-Akt in postconditioned animals and associated the upregulation of these two mediators with the enhanced NO generation shown in the same study [83]. NO-mediated upregulation of HIF-1*α* was also demonstrated by postconditioning from Rb1 [63]. In fact, other studies have also shown that NO can upregulate the rate of HIF-1*α* generation [122, 123], while activation and upregulation of HIF-1*α* have been found to protect liver from IR injury [124]. In turn, HIF-1*α* has been reported to be able to improve the actions of NO [125]. Regarding PI3K-Akt, Akt is important in the activation of e-NOS-mediated NO production and NO production following Akt-mediated e-NOS activation has been shown to convey cardioprotection [126]. The PI3K-Akt pathway has also been implicated as an important signaling mediator in liver IPC [127, 128], while it has been shown that it plays an important role in the protective action of cardiac IPostC [129]. Therefore, the Akt/e-NOS/NO/HIF-1*α* pathway could also play a role in the protective action of liver IPostC. It is also possible that milrinone, adenosine A_2_A receptor agonists, and Rb1-induced postconditioning could be associated with the activation of PI3K-Akt pathway and its downstream effectors, as shown in the relevant studies [52, 58, 63]. Finally, NO could also exert its cytoprotective effects through prevention of mitochondrial permeability transition in hepatocytes through guanylyl cyclase and cyclic guanosine monophosphate-dependent kinase signaling pathway, as shown by Kim et al. [130].

The induction of HO-1 could also be implicated in the protective role of postconditioning, as shown by Zeng et al. [46, 47]. HO-1 is upregulated by a variety of physiological and endogenous stimuli. Overexpression of HO-1 exerts cytoprotective function in a number of IR models, possibly through anti-inflammatory, antioxidant, and antiapoptotic properties, with reports of modulation of intrahepatic sinusoidal tone, improved liver microcirculation, and reduction of early oxidative burst by HO-1 and its byproducts [131–133]. HO-1 also potentiates the survival of small-for-size liver grafts [134], while some studies have suggested that IPC may exert its protective effects and suppress systemic inflammatory responses via enhanced HO-1 expression [135–138]. Additionally, it has been shown that lung IPostC attenuates lung ischemic reperfusion injury through HO-1 upregulation [139, 140]. Therefore, it could also be possible that the protective effects of hepatic postconditioning could also be mediated by upregulation of HO-1 expression [46, 47].

## 9. Conclusion

Despite the favorable effects of IPC, documented in a variety of experimental and some clinical settings, it has an obvious disadvantage: it must be initiated before the ischemic event, which is not always a feasible option. In contrast, the protection afforded by IPostC, which could be seen as an attempt for slow intermittent oxygenation through several cycles of on/off flow before permanent reperfusion, could be considered as a more appropriate choice with a theoretical clinical application value. Especially in the context of deceased donor liver transplantation, the onset of ischemia cannot be predicted and therefore postconditioning is a more appealing strategy for clinical implementation, since the onset of reperfusion is more predictable and manipulations targeting this period can be more attractive. Even in the setting of hepatectomies, preconditioning requires timely planning and is not suitable for emergency situations. However, intermittent interruptions of blood flow in the early phase of reperfusion seem to be a more suitable alternative, since the onset of reperfusion is easy to define and can be applied selectively with precisely controlled timing in patients in need for unpredictably prolonged periods of inflow occlusion.

A growing body of evidence shows that IPC and IPostC share many similarities in the mechanisms involved. However, unlike IPC, IPostC focuses on the early events of reperfusion and applies the brief episodes of ischemia at the onset of revascularization, basically constituting a variation of controlled reperfusion. It could be considered as a process that targets the first few minutes of reperfusion that modifies reperfusion injury rendering the cell and mitochondria more tolerant to the biochemical and metabolic perturbation that occurs in the transition from ischemia to reperfusion. Moreover, it has been postulated that when ischemic tissues are reperfused straight away, abrupt reperfusion flow washes out endogenous protective substances, while a slower and more controlled reperfusion with the short series of repetitive cycles of reperfusion and reocclusion maintains protective substances inside the liver parenchyma for longer.

The beneficial effects of IPostC have been documented in several experimental studies in different organs [141] and confirmed in human clinical studies in the heart [34, 35]. As explored in the current review, there have been several experimental studies of the benefits of IPostC for the liver but we were able to identify just one very recent study of its use in the clinical setting, in the context of adult cadaveric liver transplantation [48]. Unlike donor preconditioning, which is not always feasible as mentioned above, graft postconditioning in the recipient seems to be a more appealing strategy since it can be applied selectively in settings prone to greater risks for IR injury due to donor-, procurement-, or recipient-related factors and can prove useful in complex cases requiring long periods of ischemia or with marginal grafts. Based on these findings, it seems reasonable to explore the possibility of the use of IPostC in the context of clinical liver surgery beyond transplantation, especially in complex cases needing long periods of ischemia, in cases of unexpected ischemia, where there is limited clinical applicability for IPC, and during major hepatectomies, with marginal liver remnants. In the light of this, a 30% reduction of liver cell necrosis was demonstrated with the application of IPostC by Knudsen et al. [43]. If this is extrapolated to the clinical context, a necrosis reduction of this size could be crucial for patients subjected to large liver resections. Such resections can be complicated by bleeding, which is often unpredictable, and bleeding has to be controlled by clamping. In this context, IPC is not a feasible option and IPostC is the technique of choice because it can be applied after clamping to ensure the survival of as much liver parenchyma as possible. Apart from the reduction of liver cell necrosis, the favorable effect of IPostC on liver regeneration [71, 74, 75] as well as the requirement for less operative time in contrast to preconditioning techniques makes postconditioning clinically more attractive to implement in the context of extended hepatectomies.

It has been shown that the early period of reperfusion is an important period for salvaging ischemic tissue since rapid production of ROS, opening of mPTP, and fluctuations in pH develop during this period [43, 98, 101]. Moreover, results from Zhao et al. in cardiac tissues have demonstrated that manipulation of the early reperfusion phase is crucial for the protective effect of IPostC [31]. In fact, when the maneuver of IPostC was delayed for a while, instead of being applied at the very onset of reperfusion, its protective effect was lost [32]. Therefore, proper choice of timing and number of reperfusion/reocclusion cycles should be refined in future studies in the setting of liver surgery. Pharmacological inducers of postconditioning could also be useful in alleviating the metabolic, structural, and functional changes of IR injury and should be thoroughly evaluated. Future studies will be required to standardize the postconditioning procedure, to clarify its clinical impact, and to deepen its molecular understanding.

## Figures and Tables

**Table 1:** Summary of outcome of studies on ischemic postconditioning of the liver.

Study group	Year	Species	Hepatic ischemia	Ischemia time	Reperfusion time	IPostC technique	Parameters assessed	Outcome of IPostC (IPostC versus control)	Proposed mechanism
Sun et al. [37]	2004	Rat	Warm and partial	60 min	120 min	2/3/5/7 min R + 2 min I	MDA, SOD, CAT, GSH-P_X_, Bcl-2 protein, apoptotic index, and mitochondrial ultrastructure	(i) ↓MDA and apoptotic index (ii) ↑SOD, CAT, GSH-P_X_ and Bcl-2 protein, and improved mitochondrial ultrastructure	Modulation of apoptosis cascade and maintenance of integrity of mitochondrial membrane

Wu et al. [65]	2007	Rat	Warm and partial				ALT, AST, MDA, GSH, SOD, GSH-P_X_, MPO, and one-week survival	(i) ↓ALT, AST, MDA, and MPO (ii) ↑GSH, SOD, GSH-P_X_, and improved animal survival	Not addressed

Wang et al. [66]	2008	Rat	Warm, total, and cold (transplantation)	30 min of warm ischemia; 2 h of cold ischemia	3 h	(30 sec R + 30 sec I) × 3	ALT, AST, bile *γ*GT, bile glucose, histopathology, hepatocyte apoptotic activity, apoptotic-related protein Fas, and one-week survival	(i) ↓ALT, AST, ↓apoptotic activity, and Fas (ii) ↓bile *γ*GT, and bile glucose (iii) ↓inflammation and necrosis on histopathology and improved animal survival	Inhibition of apoptosis

Wang et al. [45]	2009	Rat	Cold (transplantation)	24 h	6 h	(30 sec R + 30 sec I) × 3 or 6	ALT, AST, LDH, MDA, SOD, GSH-P_X_, MPO, TNF-*α* expression, MIP-2 expression, histopathology, NO, i-NOS expression, and e-NOS expression	(i) ↓ALT, AST, and LDH (ii) ↓MDA and MPO (iii) ↓TNF-*α* and MIP-2 expression (iv) ↑SOD, GSH-P_X_, and improved hepatic morphology (v) ↑NO and i-NOS and e-NOS expression	Inhibition of neutrophil recruitment and activation, i-NOS and e-NOS-mediated NO production

Zhang et al. [38]	2009	Rat	Warm and partial	60 min	120 min	2/3/5/7 min R + 2 min I	ALT, AST, NF-*κ*B p65 expression, SOD, apoptotic index, and light and electron microscopy	(i) ↓ALT and AST (ii) ↓NF-*κ*B p65 expression and apoptotic index (iii) ↑SOD (iv) Lower degree of sinusoid congestion and neutrophilic infiltration, lower degree of disruption of nuclear and mitochondrial membranes, and lower degree of degranulation of endoplasmic reticulum	Modulation of apoptotic cascade and maintenance of mitochondrial ultrastructure and function

Teixeira et al. [40]	2009	Rat	Warm and partial	60 min	120 min	(5 sec R + 5 sec I) × 5	MDA and GST-*α*3	(i) ↓MDA (ii) Nonsignificant increase in expression of GST-*α*3	Not addressed

Dos Santos et al. [41]	2010	Rat	Warm and total	30 min	60 min	(30 sec R + 30 sec I) × 3	ALT, AST, and histopathology	(i) ↓ALT and AST (ii) Decreased congestion and hepatocyte degeneration	Not addressed

Zeng et al. [46]	2010	Rat	Cold (transplantation)	24 h	6 h	(60 sec R + 60 sec I) × 6	ALT, AST, MDA, SOD, HO-1 expression, and histopathology	(i) ↓ALT, AST, and MDA (ii) ↑SOD and HO-1 expression (iii) Decreased vacuolization, sinusoidal congestion, and hepatocyte necrosis	Upregulation of HO-1 expression

Knudsen et al. [71]	2010	Rat	Warm and total	30 min	30 min	(30 sec R + 30 sec I) × 3	ALT and gene expression analysis after RNA extraction	(i) ALT not different from control and upregulation of genes involved in angiogenesis	Not addressed

Guo et al. [83]	2011	Mouse	Warm and partial	60 min	2/4/12 h	(10 sec R + 10 sec I) × 3	ALT, MDA, SOD, NO, e-NOS, TNF-*α*, ICAM-1, HIF-1*α*, PI3K-Akt, and histopathology	(i) ↓ALT, MDA, TNF-*α*, and ICAM-1 (ii) ↑SOD, NO, and e-NOS (iii) ↑HIF-1*α* and PI3K-Akt (iv) Lower scores of cytoplasmic vacuolization, sinusoidal congestion, and hepatocyte necrosis	Suppression of proinflammatory mediators and adhesion molecules, e-NOS-mediated NO production through PI3K-Akt, and upregulation of HIF-1*α* through NO

Knudsen et al. [70]	2011	Rat	Warm and total	30 min	30 min	(30 sec R + 30 sec I) × 3	ALT, HIF-1*α*, and VEGF	(i) ALT not different from control and lack of upregulation of HIF-1*α* and VEGF	Not addressed

Zeng et al. [47]	2011	Rat	Cold (transplantation)	24 h	6 h	(60 sec R + 60 sec I) × 6	ALT, AST, MDA, SOD, HO-1 expression, histopathology, and electron microscopic examination	(i) ↓ALT, AST, and MDA (ii) ↑SOD and HO-1 expression (iii) Decreased vacuolization, sinusoidal congestion and hepatocyte necrosis, and decreased evidence of chromatin damage and mitochondrial disruption	Upregulation of HO-1 expression

Lin et al. [42]	2012	Rat	Warm and partial	45 min	4 h	(60 sec R + 60 sec I) × 3	ALT, apoptotic index, 4-HNE modified protein, cytochrome c release from mitochondria, and change of mitochondrial membrane potential	(i) ↓ALT, apoptotic count, and 4-HNE modified protein (ii) Attenuated cytochrome c release from mitochondria and preservation of mitochondrial membrane potential	Inhibition of mitochondrial permeability transition pore opening, preservation of the electrochemical gradient across the inner mitochondrial membrane, and inhibition of release of proapoptotic solutes like cytochrome c

Song et al. [67]	2012	Mouse	Warm and partial	30 min	60 min	(30 sec R + 30 sec I) × 3	ALT, AST, TNF-*α*, IL-1*β*, T-AOC, MDA, SOD, CAT, GSH-P_X_, T-NOS, i-NOS, HIF-1*α*, and VEGF	(i) ↓ALT, AST, TNF-*α*, IL-1*β*, and MDA (ii) ↑T-AOC, SOD, CAT, GSH-P_X_, T-NOS, i-NOS, HIF-1*α*, and VEGF	Induction of hepatic own defensive mechanism for tissue adaptation in oxygen-deficient environments

Knudsen et al. [74]	2012	Rat	Warm and total	30 min	30 min	(30 sec R + 30 sec I) × 3	ALT, gene expression analysis after RNA extraction, and quantitative real-time PCR	(i) ALT not different from control (ii) Upregulation of genes involved in DNA binding and transcription, cellular membrane function, and apoptosis and metabolic processes	Not addressed

Knudsen et al. [43]	2013	Rat	Warm and partial	60 min	4/24 h	(30 sec R + 30 sec I) × 3	ALT, TNF-*α*, IL-6, NVR, and apoptotic cell number	(i) ALT not different from control, variable kinetics of IL-6, and TNF-*α* below detection limit at all time points (ii) ↓NVR (iii) ↓apoptotic cell profiles (insignificantly)	

Young et al. [75]	2014	Prepubertal rat	Warm and total	30 min	24 h	(30 sec R + 30 sec I) × 2	ALT, AST, PCNA, and regenerated liver mass	(i) ↓AST and ALT (ii) Improved liver regeneration	Not addressed

Yoon et al. [39]	2015	Rat	Warm and partial	60 min	120 min	2/3/5/7 min R + 2 min I	ALT, AST, MDA, and survivin	(i) No significant difference in ALT, AST, and MDA (ii) Increased expression of survivin	Inhibition of apoptosis

Ricca et al. [48]	2015	Man	Cold (transplantation)	Not standard	120 min for reperfusion biopsies	(60 sec R + 60 sec I) × 3 at arterial reperfusion	AST (peak postop levels), EGD, histopathology, evidence of apoptosis or autophagy, postop morbidity and mortality, and one-year patient and graft survival	(i) Less severe injury on histology (ii) Increased autophagy (iii) No difference in median postop AST, EGD, apoptosis, postop morbidity and mortality, and one-year patient and graft survival	Not addressed

R: reperfusion; I: ischemia; IPostC; ischemic postconditioning; MDA: malondialdehyde; SOD: superoxide dismutase; CAT: catalase; GSH-P_X_: glutathione peroxidase; ALT: alanine aminotransferase; AST: aspartate aminotransferase; MPO: myeloperoxidase; *γ*GT: gamma-glutamyltransferase; LDH: lactate dehydrogenase; TNF-*α*: tumor necrosis factor-alpha; MIP-2: macrophage inflammatory protein-2; NO: nitric oxide; i-NOS: inducible NO synthase; e-NOS: endothelial NO synthase; NF-*κ*B: nuclear factor-kappa B; GST-*α*3: glutathione-s-transferase-*α*-3; ICAM-1: intercellular adhesion molecule-1; HIF-1*α*: hypoxia inducible factor 1-alpha; PI3K-Akt: phosphatidylinositol 3-kinase; VEGF: vascular endothelial growth factor; 4-HNE: 4-hydroxy-2-nonenal; T-AOC: total antioxidant capacity; T-NOS: total NOS; PCR: polymerase chain reaction; NVR: necrotic volume ratio; PCNA: proliferating cell nuclear antigen; EGD: early graft dysfunction.

**Table 2:** Summary of outcome of studies on pharmacological postconditioning of the liver.

Study group	Year	Species	Hepatic ischemia	Ischemia time	Reperfusion time	Pharmacological inducer of postconditioning	Parameters assessed	Outcome of postconditioning (postconditioning versus control)	Proposed mechanism
Guo et al. [63]	2011	Mouse	Warm and partial	60 min	2/4/12 h	Ginsenoside Rb1	ALT, MDA, SOD, NO, e-NOS, TNF-*α*, ICAM-1, HIF-1*α*, PI3K-Akt, and histopathology	(i) ↓ALT, MDA, TNF-*α*, and ICAM-1 (ii) ↑SOD, NO, and e-NOS (iii) ↑HIF-1*α* and PI3K-Akt (iv) Lower scores of cytoplasmic vacuolization, sinusoidal congestion, and hepatocyte necrosis	Suppression of proinflammatory mediators and adhesion molecules, e-NOS mediated NO production through PI3K-Akt, and upregulation of HIF-1*α* through NO

Dal Ponte et al. [58]	2011	Rat	Hepatocyte culture and cold storage (in vitro)/warm partial ischemia (in vivo)	24 h cold ischemia/60 min warm ischemia	120 min	A_2_A receptor agonist	Hepatocyte viability, ALT, PI3K-Akt, and histopathology	(i) ↓ALT (ii) ↑hepatocyte viability and PI3K-Akt (iii) Fewer necrotic areas on histological examination	Activation of PI3K-Akt pathway

Beck-Schimmer et al. [56]	2012	Man	Warm and total	At least 30 min	Not standard	Sevoflurane	AST, ALT (peak postop levels), postop complications, and length of hospital stay	(i) ↓AST (ii) ALT not different from control (iii) ↓incidence of complications and shorter hospital stay	Not addressed

Shawky et al. [64]	2012	Rat	Warm and partial	45 min	120 min	Recombinant human erythropoietin	AST, ALT, caspase-9 activity, Fas ligand expression, antiapoptotic Bcl-xL/apoptotic Bax ratio, and histopathology	(i) ↓AST and ALT (ii) No change in Fas ligand expression (iii) Improved histology scores (iv) Preconditioning with rhEPO more effective than postconditioning in the reduction of caspase-9 activity and the increase of antiapoptotic Bcl-xL/apoptotic Bax ratio	Modulation of apoptosis cascade

Tian et al. [53]	2013	Rat	Warm and partial	60 min	120 min	Diazoxide	AST, ALT, pkc-*ε*, Bcl-2, cytochrome c, and caspase-3	(i) ↓AST and ALT (ii) ↑pkc-*ε* and Bcl-2 (iii) ↓cytochrome c and caspase-3	Upregulation of pkc-*ε*, opening of mito-K_ATP_ channels, and inhibition of the apoptotic pathway

Toyoda et al. [52]	2014	Rat	Warm and partial	60 min	5 h	Milrinone	AST, ALT, LDH, histopathology, and apoptotic score	(i) ↓AST, ALT, and LDH (ii) Improved histology scores and lower apoptotic rate	Activation of PI3K-Akt pathway and upregulation

ALT: alanine aminotransferase; MDA: malondialdehyde; SOD: superoxide dismutase; NO: nitric oxide; e-NOS: endothelial NO synthase; TNF-*α*: tumor necrosis factor-alpha; ICAM-1: intercellular adhesion molecule-1; HIF-1*α*: hypoxia inducible factor 1-alpha; PI3K-Akt: phosphatidylinositol 3-kinase; AST: aspartate aminotransferase; pkc-*ε*: protein kinase c-epsilon; LDH: lactate dehydrogenase.

## Abbreviations

IR: Ischemia-reperfusion
ROS: Reactive oxygen species
NO: Nitric oxide
IPC: Ischemic preconditioning
IPostC: Ischemic postconditioning
MDA: Malondialdehyde
SOD: Superoxide dismutase
CAT: Catalase
GSH-P_X_: Glutathione peroxidase
TUNEL: Terminal deoxynucleotidyl transferase dUTP nick end labeling
ALT: Alanine aminotransferase
AST: Aspartate aminotransferase
NF-*κ*B: Nuclear factor-kappa beta
4-HNE: 4-Hydroxy-2-nonenal
NVR: Necrotic volume ratio
LDH: Lactate dehydrogenase
MPO: Myeloperoxidase
RT-PCR: Reverse transcriptase-polymerase chain reaction
TNF-*α*: Tumor necrosis factor-alpha
MIP-2: Macrophage inflammatory protein-2
i-NOS: Inducible NO synthase
e-NOS: Endothelial NO synthase
HO-1: Heme oxygenase-1
cAMP: Cyclic adenosine monophosphate
PKA: Protein kinase A
PI3K-Akt: Phosphatidylinositol 3-kinase
mito-K_ATP_: ATP-dependent mitochondrial potassium
pkc-*ε*: Protein kinase c-epsilon
Rb1: Ginsenoside Rb1
rhEPO: Human recombinant erythropoietin
HIF-1*α*: Hypoxia inducible factor 1-alpha
VEGF: Vascular endothelial growth factor
ICAM-1: Intercellular adhesion molecule-1.
